# Rutaecarpine Ameliorates Pressure Overload Cardiac Hypertrophy by Suppression of Calcineurin and Angiotensin II

**DOI:** 10.1155/2021/8857329

**Published:** 2021-01-12

**Authors:** Shujun Li, Bo Huang, Changfei Zhou, Jingshan Shi, Qin Wu, Qingsong Jiang

**Affiliations:** ^1^Department of Burn and Plastic Surgery, The first Affiliated Hospital of Zunyi Medical University, Zunyi, Guizhou 563000, China; ^2^Key Laboratory of Basic Pharmacology of Ministry of Education and Joint International Research Laboratory of Ethnomedicine of Ministry of Education, Zunyi Medical University, Zunyi, Guizhou 563000, China; ^3^Department of Pharmacology, Chongqing Key Laboratory of Biochemistry and Molecular Pharmacology, Chongqing Medical University, Chongqing 400016, China

## Abstract

Cardiac hypertrophy is a major pathological process to result in heart failure and sudden death. Rutaecarpine, a pentacyclic indolopyridoquinazolinone alkaloid extracted from *Evodia rutaecarpa* with multiple pharmacological activities, yet the underlying protective effects and the mechanisms on cardiac hypertrophy remain unclear. This study aimed to evaluate the potential effects of rutaecarpine on pressure overload cardiac hypertrophy. Cardiac hypertrophy in rat was developed by abdominal aortic constriction (AAC) for 4 weeks, which was improved by rutaecarpine supplementation (20 or 40 mg/kg/day, i.g.) for another 4 weeks. The level of angiotensin II was increased; the mRNA expression and the activity of calcineurin in the left ventricular tissue were augmented following cardiac hypertrophy. Rutaecarpine administration decreased angiotensin II content and reduced calcineurin expression and activity. Noteworthily, in angiotensin II-induced cardiomyocytes, rutaecarpine ameliorated the hypertrophic effects in a dose-dependent manner and downregulated the increased mRNA expression and activity of calcineurin. In conclusion, rutaecarpine can improve cardiac hypertrophy in pressure overload rats, which may be related to the inhibition of angiotensin II-calcineurin signal pathway.

## 1. Introduction

Cardiac hypertrophy is considered as a compensatory response to maintain cardiac output during various physiological and pathological conditions. However, prolonged hypertrophy often leads to decompensation, resulting in heart failure and sudden death. Many factors including mechanical stress and neurohumoral stimulation induce cardiac hypertrophy [1]. Among them, angiotensin II has been identified as one of the most powerful stimuli in inducing cardiac hypertrophy. It has been reported that angiotensin II-activated Ca^2+^ signaling pathway initiated the progress of cardiomyocytes hypertrophy. Angiotensin II activates G protein-dependent signaling pathways in cardiomyocytes that evokes Ca^2+^ entry [2]. The sustained intracellular Ca^2+^ concentration ([Ca^2+^]_i_) increase induces pathological myocardial hypertrophy through activation of Ca^2+^-dependent signaling pathway such as Ca^2+^-calcineurin, which directly participates in several extracellular signal pathways causing myocardial hypertrophy.

*Evodia rutaecarpa* (Wu-Chu-Yu), a traditional Chinese herb, has been used to treat various diseases for centuries. Rutaecarpine, one of the main bioactive components of *Evodia rutaecarpa* and other related herbs, is a pentacyclic indolopyridoquinazolinone alkaloid with multiple pharmacological activities, including anticoagulation, vasodilation, and anticholinesterase [3]. Rutaecarpine has multifactorial cardiovascular actions, such as cardiotonic responses, vasodilatory and blood-pressure lowering effects, endothelium protection, and antiplatelet activation. Therefore, it can be used to treat various cardiovascular diseases, including atherosclerosis, myocardial injury, and hypertension [4]. It can also attenuate ventricular remodeling in rats induced by isoprenaline or hypoxia [5, 6]. Recently, Zeng et al. found that rutaecarpine could prevent hypertensive cardiac hypertrophy [7]. However, little is known about the potential therapeutic effect of rutaecarpine on pressure overload cardiac hypertrophy. It has been found that some mechanisms are involved in the antihypertrophic effect of rutaecarpine, such as the stimulation of calcitonin gene-related peptide (CGRP) [5, 6, 8] and the inhibition of NADPH oxidase 4 (Nox4) reactive oxygen species (ROS) via the disintegrin and metalloproteinase-17 (ADAM17) pathway [7]. In addition, rutaecarpine could inhibit angiotensin II-induced proliferation of vascular smooth muscle cells and senescence of endothelial progenitor cells [8, 9]. Nevertheless, the underlying mechanisms of rutaecarpine on angiotensin II-related signal pathways in cardiac hypertrophy have not been fully clarified, especially the effect on angiotensin II-calcineurin remains unknown.

In the present study, therefore, the therapeutic effect of rutaecarpine on pressure overload cardiac hypertrophy and the possible mechanisms related to angiotensin II-calcineurin were investigated *in vivo* and *in vitro*.

## 2. Materials and Methods

### 2.1. Chemicals and Reagents

Rutaecarpine (MW: 287.32; purity: HPLC ≥ 98%) was purchased from Nanjing Zelang Biotechnology Co., Ltd. (Jiangsu, China); BCA assay kit was purchased from Beyotime Biotechnology Co. Ltd. (Jiangsu, China); *α*-actin and calcineurin antibodies were purchased from Abcam (Cambridge, MA, USA); 4′, 6-diamidino-2-phenylindole (DAPI) was purchased from Sigma-Aldrich (St. Louis, MO, USA); angiotensin II radioimmunoassay kit was purchased from Beijing North Institute of Biological Technology (Beijing, China); calcineurin activity kit was purchased from Nanjing Jiancheng Biology Engineering Institute (Shanghai, China); RT-qPCR primers were custom-synthesized and purified by Invitrogen (Shanghai, China). All other reagents were from commercial suppliers and were of standard biochemical quality.

### 2.2. Induction of Cardiac Hypertrophy In Vivo

Healthy adult male Sprague-Dawley (SD) rats weighing between 190 and 220 g were purchased from Daping Experimental Animal Center of the Third Affiliated Hospital of Army Medical University (Chongqing, China, Animal qualified certificate: SCXK 2012-0005) and provided humanitarian care. Animals were housed in a temperature-controlled room (25 ± 2°C) with a 12 h light/dark cycle and were given free access to standard laboratory pellet diet and water. All experimental procedures were in accordance with the NIH Guide for Care and Use of Laboratory Animals and approved by the Animal Experiment Ethics Committee of Zunyi Medical University.

Pressure overload was produced by abdominal aortic constriction (AAC), which has primarily been used as a model of cardiac hypertrophy [9]. Briefly, rats (*n* = 46) were anesthetized using 5% pentobarbital (60 mg/kg, ip) and the aorta was exposed through a midline abdominal incision. For the banding model, a blunt 21-gauge needle was placed adjacent to the abdominal aorta between the renal arteries just below the renal bifurcations, and a ligature was tightened around the aorta and adjacent needle. The sham procedure for the control rats included injection of the same dose of combination anesthesia, an incision of approximately the same size, and the placement of a loosely tied ligature at the same position on the abdominal aorta. The muscular layer was sutured, followed by the abdominal skin suture, and the animals were isolated in a cage for recovery. Each rat was given penicillin 120 000 units i.p. for 3 days to avoid infection.

Similar to the results of our pervious [10] and other studies [11, 12], the left ventricular hypertrophy developed on the 4th week after AAC in rats, which was confirmed by pathological observation and atrial natriuretic factor (ANF) mRNA expression in six randomly selected AAC rats. The remaining AAC rats, that is, cardiac hypertrophy rats, were then randomly assigned to three groups (*n* = 8 per group), including untreated AAC rats (Model) and AAC rats treated with rutaecarpine 20 or 40 mg/kg/day i.g., respectively. Rutaecarpine was formulated freshly using 1% carboxy methyl cellulose (CMC) in distilled water and administered orally at different doses for another 4 weeks. The sham-operated rats and cardiac hypertrophy rats were given an equal volume of 1% CMC solution for 4 weeks.

Rats were sacrificed under anesthesia. Body weight (BW) was recorded and heart was separated into the left ventricle with septum (LV + S) and the right ventricle (RV) and weighed separately. Finally, the (LV + S)/RV and (LV + S)/BW were calculated to evaluate cardiac hypertrophy.

### 2.3. Pathological Examination in Left Ventricle

The left ventricular tissue was fixed with 10% formaldehyde solution for 48 h, then dehydrated with graded alcohol, and then embedded in paraffin and 3-5 *μ*m thick sections were stained with H&E. The histopathological changes were examined with an optical microscopic (BX-43, Olymous Co. Ltd., Tokyo, Japan).

### 2.4. Induction of Cardiomyocytes Hypertrophy In Vitro

Ventricular myocytes from 1- to 3-day-old SD rats were prepared and cultured for 48 h in Dulbecco's modified Eagle's medium (DMEM) containing 20% fetal bovine serum and 0.1 mmol/L 5′-bromodeoxyuridine. The seeding density was about 1 × 10^5^ cells/mL for measuring cell diameters or 1 × 10^6^ cells/mL for evaluating cellular total protein content by BCA kits. The medium was replaced by serum-free DMEM for a further 48 h before pharmacological treatment. Angiotensin II at 1 *μ*mol/L was used to stimulate the cardiomyocytes. The antihypertrophic effects of rutaecarpine (dissolved in DMSO of the final concentration less than 0.1%) from 0.1 to 10 *μ*mol/L were studied.

Immunofluorescence staining was performed to identify the cardiomyocytes by *α*-actin antibody (red fluorescence) and DAPI (blue fluorescence). Cellular hypertrophy was evaluated by the increase of cardiomyocytes diameter and protein level. The diameter of single cells was measured by a digital image analysis system (Leica QwinV3, Leica Microsystems Ltd., USA). Five random fields (with approximately 10 to 15 cells per field) from every sample were averaged and expressed as *μ*m/cell. Collected cardiomyocytes protein was extracted with RIPA lysate and determined by BCA assay. The protein concentration per 10^6^ cells was detected to calculate the amount of protein per cell. All experiments were repeated six times.

### 2.5. Measurement of ANF and Calcineurin mRNA by RT-qPCR

According to the instructions, Trizol was used to extract total RNA from left ventricle tissue or cultured cardiomyocytes. The real-time PCR reaction procedure (95°C for 30 s; 95°C for 5 s, 60°C for 30 s, 40 cycles) was carried out on a RT-qPCR instrument (Bio-Rad CFX96, CA, USA). The primer sequences used were shown in Table 1 and *β*-actin was used as an internal reference gene. The Ct value was used as the statistical parameter and the expression fold of mRNA was expressed as 2^−ΔΔCt^. All experiments were repeat four times for each group.

### 2.6. Measurement of Angiotensin II Level and Calcineurin Activity

The left ventricle tissue or cultured cardiomyocytes were homogenized with RIPA lysate and finally centrifuged at 4°C for 20 min at 13, 000 ×g. The protein concentration in the supernatant was detected with a BCA assay kit. Meanwhile, the level of angiotensin II in tissue was detected with an angiotensin II radioimmunoassay kit. The activity of calcineurin was detected with a calcineurin assay kit and was expressed as Pi produced by the calcineurin decomposition of substrate PNPP per hour from per mg of total protein (*μ*mol pi/h mg pro). All experiments were repeated six times.

### 2.7. Statistical Analysis

Results were expressed as mean ± SD and statistical analysis was performed using SPSS 20.0. The mean value among groups was analyzed by one-way analysis of variance (ANOVA) and *P* < 0.05 was considered statistically significant.

## 3. Results

### 3.1. Effects of Rutaecarpine on AAC-Induced Cardiac Hypertrophy

**Figure 1:**
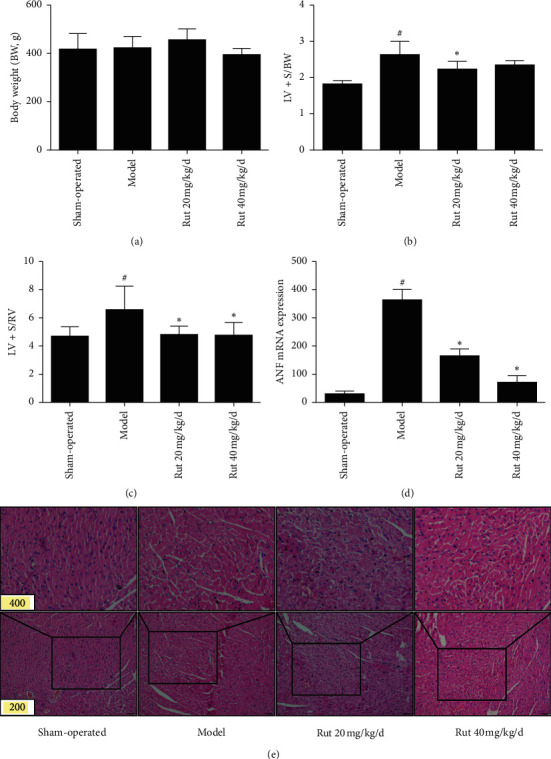
Effects of rutaecarpine on left ventricular hypertrophy induced by AAC. (a) Body weight (BW) (*n* = 8); (b) (LV + S)/BW (*n* = 8); (c) (LV + S)/RV (*n* = 8), (d) ANF mRNA expression (*n* = 4), and (e) myocardial morphological changes by H&E staining. ^#^*P* < 0.05 versus sham-operated; ^*∗*^*P* < 0.05 versus model. LV + S: left ventricle with septum; RV: right ventricle.

Figure 1 showed that there was no significant change in BW in every group (*P* > 0.05). However, (LV + S)/BW, (LV + S)/RV, and ANF mRNA expression significantly increased in model group (*P* < 0.05), in comparison with sham-operated group. The pathomorphology of the left ventricle showed that larger cardiomyocytes and irregular and disruptive fiber in model group were observed. Administration of rutaecarpine at 20 or 40 mg/kg/d could significantly improve cardiac hypertrophy in AAC-induced rats, which decreased (LV + S)/BW, (LV + S)/RV, and ANF mRNA expression in a dose-dependent manner and ameliorated the pathological changes of the left ventricle (Figure 1).

### 3.2. Effects of Rutaecarpine on Angiotensin II Level, Calcineurin mRNA Expression, and Activity in Left Ventricle of AAC-Induced Rats

**Figure 2:**
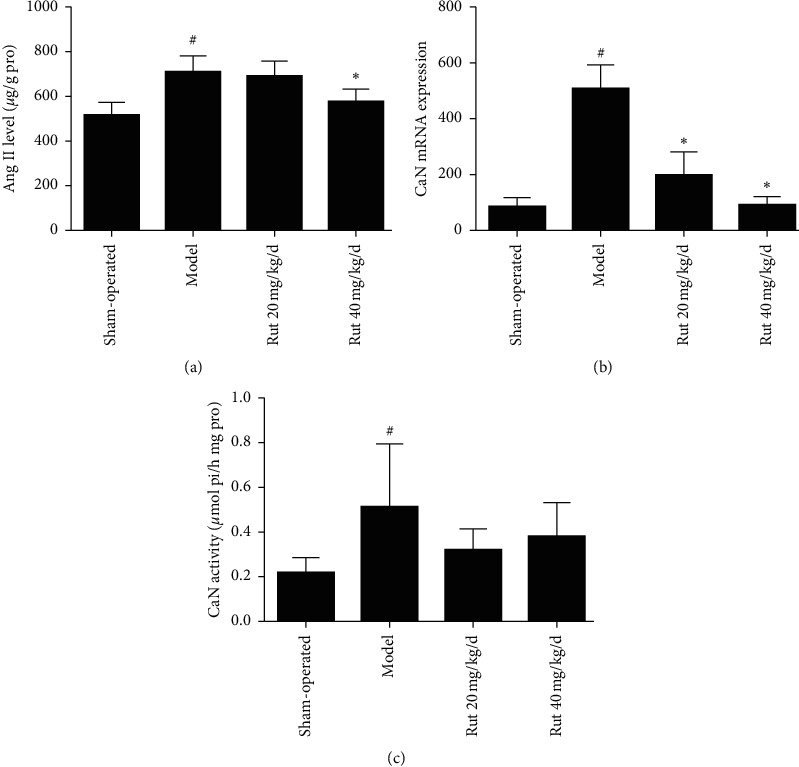
Effects of rutaecarpine (Rut) on angiotensin II (Ang II) level. (a) (*n* = 6) and calcineurin (CaN) mRNA expression, (b) (*n* = 4) and CaN activity, and (c) (*n* = 6) in left ventricle in AAC-induced rat. ^#^*P* < 0.05 versus sham-operated; ^*∗*^*P* < 0.05 versus model.

The level of angiotensin II, the mRNA expression, and the activity of calcineurin in the left ventricle increased significantly in AAC-induced rats. Rutaecarpine administration decreased the elevated angiotensin II and calcineurin in the model rats (*P* < 0.05) (Figure 2).

### 3.3. Effects of Rutaecarpine on Cardiomyocytes Hypertrophy Induced by Angiotensin II

**Figure 3:**
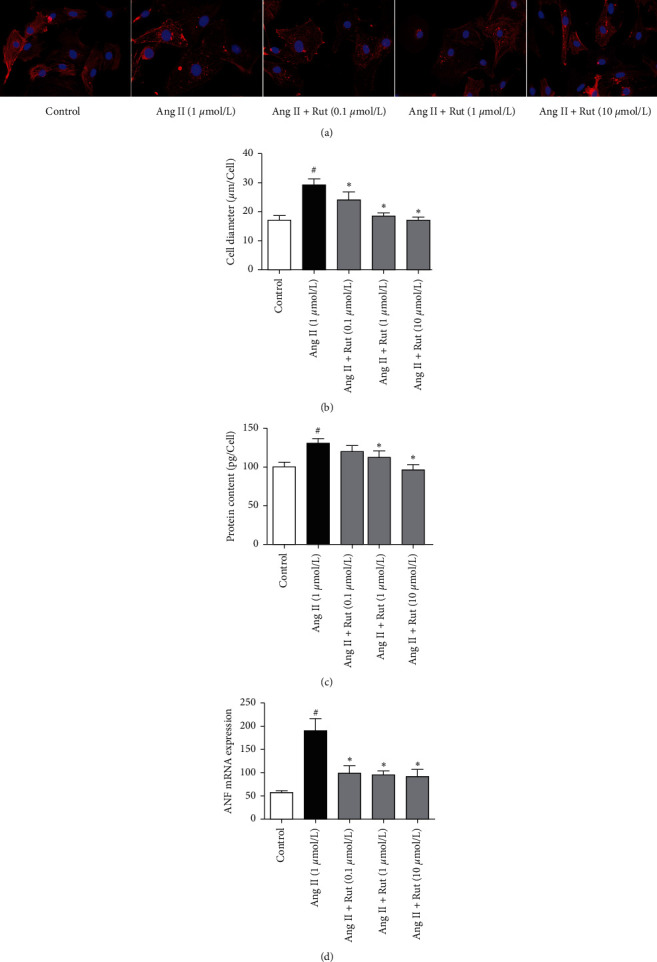
Effects of rutaecarpine (Rut) on hypertrophic cardiomyocytes induced by angiotensin II (Ang II). (a) Morphological changes of cardiomyocytes by immunofluorescence (400×), (b) cell diameter (*n* = 6), (c) protein content (*n* = 6), and (D) (*n* = 4). ^#^*P* < 0.05 versus control; ^*∗*^*P* < 0.05 versus Ang II.

Angiotensin II (1 *μ*mol/L) stimulation caused significant cardiomyocytes hypertrophy following 48 h incubation. Cell diameter and total protein content increased by 75.0% and 31.2%, respectively, while ANF mRNA increased 2.3-fold compared with the control group (*P* < 0.05) (Figure 3). Treatment with rutaecarpine (from 0.1 to 10 *μ*mol/L) significantly relieved the changes induced by angiotensin II in a concentration-dependent manner (*P* < 0.05).

### 3.4. Effects of Rutaecarpine on Calcineurin mRNA Expression and Activity in Angiotensin II-Induced Hypertrophic Cardiomyocytes

**Figure 4:**
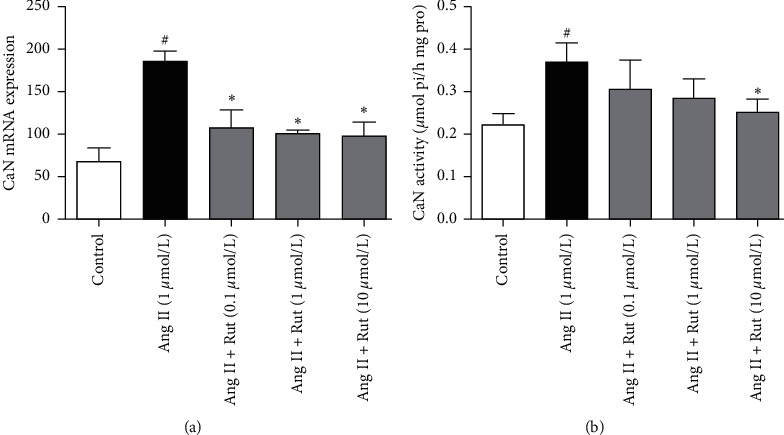
Effects of rutaecarpine (Rut) on calcineurin (CaN) level and CaN activity in angiotensin II- (Ang II-) induced cardiomyocytes. (a) mRNA expression (*n* = 4) and (b) CaN activity (*n* = 6). ^#^*P* < 0.05 versus control; ^*∗*^*P* < 0.05 versus Ang II.

In angiotensin II-conditioned cardiomyocytes, calcineurin mRNA increased by 167.9%, while calcineurin activity increased by 56.5% after 48 h incubation (*P* < 0.05). Treatment with rutaecarpine (from 0.1 to 10 *μ*mol/L) significantly relieved the changes induced by angiotensin II in a concentration-dependent manner (*P* < 0.05) (Figure 4).

## 4. Discussion

Cardiac hypertrophy is an adaptive response of the heart to pressure or volume overload, myocardial infarction, and other cardiovascular stimuli [13]. Hypertension is the most important background of cardiac overload. In the response to long-standing arterial hypertension, heart function is maintained through enlarged cardiomyocytes and increased protein synthesis, accompanied by the reactivation of the fetal gene expression, such as ANF, which are the characteristics of cardiac hypertrophy. The abdominal aorta is constricted to increase cardiac pressure overload and induce cardiac hypertrophy, which is more clinically relevant and similar to the human form of the disease [9, 14]. Numerous studies have found that the left ventricular hypertrophy was developed on the 4th week after AAC in rats, which was similar to the results in the present study (data not shown). In the end of the experiment, the AAC-operated rats developed significantly cardiac hypertrophy, which manifested as increased (LV + S)/BW, (LV + S)/RV, and ANF mRNA expression, as well as the histopathological changes of left ventricle. Rutaecarpine has protective effects in different cardiac hypertrophy rats [8]. In hypertensive cardiac hypertrophy, rutaecarpine has also shown a preventive role [7]. Our results found that rutaecarpine treatment could improve the pathological changes and decrease the mRNA expression of ANF in a dose-dependent way in AAC-induced hypertrophy. The results suggested that rutaecarpine has also a beneficial therapeutic effect against ventricular hypertrophy induced by pressure overload.

Mechanical stress and neurohumoral mechanisms are known to stimulate the initiation of myocardial hypertrophy. Angiotensin II, an important active peptide from the renin-angiotensin system, has been defined as a powerful stimulus to induce cardiac hypertrophy [15]. Similar to the studies, angiotensin II was also increased in left ventricular tissue in AAC-induced rats. In cultured primary cardiomyocytes cultures, angiotensin II caused significant hypertrophy by increasing cell diameter, protein content, and ANF mRNA expression. Rutaecarpine is effective against angiotensin II-induced rat vascular smooth muscle cells proliferation [16]. It can also counteract angiotensin II-induced endothelial progenitor cells senescence [17]. Our results showed that the antihypertrophic effect of rutaecarpine was accompanied by the decrease of angiotensin II level in AAC-operated rats. In angiotensin II-induced primary cardiomyocytes, rutaecarpine also showed significant antihypertrophic effects in a concentration-dependent way. Combined with the results from *in vivo* and *in vitro*, the effect of rutaecarpine may be related to the inhibition of angiotensin II.

It has been reported that angiotensin II-activated Ca^2+^ signaling pathway initiated the progress of cardiomyocytes hypertrophy [18, 19]. Angiotensin II activates AT_1_ receptor induces Ca^2+^ influx via complex interacting signaling pathways involving G protein-mediated activation of phospholipase C (PLC), which in turn generates diacylglycerol (DAG) and inositol 1,4,5-trisphosphate (IP_3_) that are responsible for sustained increase in [Ca^2+^]_i_ [20]. The increasing [Ca^2+^]_i_ stimulates Ca^2+^-related signal pathway, for example, calcineurin. The activation of calcineurin in cardiac cells is sufficient to induce cardiac hypertrophy [15]. However, little is known about the effect of rutaecarpine on angiotensin II-calcineurin pathway. In the present study, the mRNA expression and the activity of calcineurin were up-regulated in AAC-model rats and in angiotensin II-induced cardiomyocytes. Rutaecarpine treatment could reduce the levels of calcineurin in AAC-operated model rats; notably, rutaecarpine could also downregulate the increases of calcineurin in angiotensin II-induced cardiomyocytes. These results suggested that the antihypertrophic effect of rutaecarpine is related to reducing the activity of angiotensin II-calcineurin signal pathway.

Taken together, the present study showed that rutaecarpine has potential therapeutic effect on pressure overload cardiac hypertrophy induced by AAC-operated rats, which may be, at least partly, related to the downregulation of calcineurin via the inhibition of angiotensin II pathway.

## Figures and Tables

**Table 1:** Primer sequences for real-time quantitative RT-PCR.

Gene	Forward primer (5′-3′)	Reverse primer (5′-3′)
ANF	TGACAGGATTGGAGCCCAGAG	TCGAGCAGATTTGGCTGTTATCTTC
CaN	CTGAGATGCTGGTAAACGTCCTGA	TGCTCGGATCTTGTTCCTGATG
*β*-actin	GGAGATTACTGCCCTGGCTCCTA	GACTCATCGTACTCCTGCTTGCTG

## Data Availability

The data used to support the findings of this study are included within the article.

## Abbreviations

AAC: Abdominal aortic constriction
ANF: Atrial natriuretic factor
Ang II: Angiotensin II
BW: Body weight
BSA: Bull serum albumin
CaN: Calcineurin
LV + S: Left ventricle with septum
RV: Right ventricle
Rut: Rutaecarpine.
